# The functional and anatomical dissection of somatosensory subpopulations using mouse genetics

**DOI:** 10.3389/fnana.2014.00021

**Published:** 2014-04-22

**Authors:** Claire E. Le Pichon, Alexander T. Chesler

**Affiliations:** ^1^National Institute of Neurological Disorders and Stroke, National Institutes of HealthBethesda, MD, USA; ^2^Intramural Pain Program, Section on Sensory Cells and Circuits, National Center for Complementary and Alternative Medicine, National Institutes of HealthBethesda, MD, USA

**Keywords:** somatosensation, pain, nociception, TRP channel, touch, thermodetection, itch, sensory neuron

## Abstract

The word somatosensation comes from joining the Greek word for body (soma) with a word for perception (sensation). Somatosensory neurons comprise the largest sensory system in mammals and have nerve endings coursing throughout the skin, viscera, muscle, and bone. Their cell bodies reside in a chain of ganglia adjacent to the dorsal spinal cord (the dorsal root ganglia) and at the base of the skull (the trigeminal ganglia). While the neuronal cell bodies are intermingled within the ganglia, the somatosensory system is in reality composed of numerous sub-systems, each specialized to detect distinct stimuli, such as temperature and touch. Historically, somatosensory neurons have been classified using a diverse host of anatomical and physiological parameters, such as the size of the cell body, degree of myelination, histological labeling with markers, specialization of the nerve endings, projection patterns in the spinal cord and brainstem, receptive tuning, and conduction velocity of their action potentials. While useful, the picture that emerged was one of heterogeneity, with many markers at least partially overlapping. More recently, by capitalizing on advances in molecular techniques, researchers have identified specific ion channels and sensory receptors expressed in subsets of sensory neurons. These studies have proved invaluable as they allow genetic access to small subsets of neurons for further molecular dissection. Data being generated from transgenic mice favor a model whereby an array of dedicated neurons is responsible for selectively encoding different modalities. Here we review the current knowledge of the different sensory neuron subtypes in the mouse, the markers used to study them, and the neurogenetic strategies used to define their anatomical projections and functional roles.

## Introduction

We can all appreciate the richness of sensation that originates from within even a small patch of skin thanks to the neurons of the somatosensory system. The peripheral targets of somatosensory neuron afferents span from the tips of your toes to the inner organs of your body where they report on the presence of a striking diversity of stimuli. The pleasant feeling of a breeze on a summer's day might involve the activation of fibers sensitive to cooling but also perhaps the silencing of warm-activated fibers. The gentle mechanical forces generated by the wind might stimulate fibers that sense the bending of single hairs or subtle pressure changes across the surface of your skin. Now imagine the breeze stops and the baking sun heats up that same piece of skin. Suddenly a different class of fibers is recruited—thermosensitive nociceptors. These fibers, whose activation can also result in the stinging sensation from a paper cut, initiate signals that we interpret as pain.

Such diversity of sensations underlies the somatosensory system's myriad of functions. For example, we easily discriminate between gradations of mechanical stimuli ranging from a gentle caress to sharp stabbing or pinching. The same is true for thermal detection where cooling or warming can be pleasurable while searing heat or extreme cold are unbearable. In addition to differentiating between innocuous and noxious stimuli, the activity of somatosensory neurons also reports on the state of the body. Alterations in nociceptor thresholds during injury and inflammation from tissue damage result in hypersensitivity. Thus, if our same patch of skin (to which we forgot to apply sunblock) is overexposed to sunlight and incurs UV damage, gentle touch suddenly becomes painful, a process referred to as mechanical *allodynia*, and it also becomes much more sensitive to heat, a process also known as thermal *hyperalgesia*.

Such acuity begs the question—how are diverse stimuli detected and encoded by the somatosensory system? Or stated slightly differently—how many sensory neuron subtypes exist and what are their relative functions? In this review we examine recent approaches that have been taken to address these questions. First, we will briefly summarize the general anatomy and physiological properties of sensory neurons as they have been traditionally classified. Then, we will highlight several recent genetic strategies that have begun to elucidate the different classes of sensory neurons and their functional roles.

### General features of the peripheral somatosensory system

**Figure 1:**
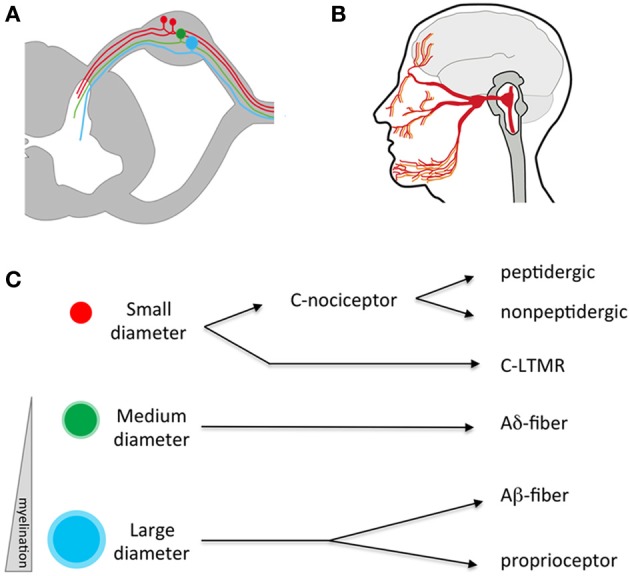
**Anatomy of the somatosensory system. (A)** Somatosensory neuron cell bodies reside outside the spinal cord in the dorsal root ganglia (DRG). They have a single process that splits, sending an afferent projection to the periphery and an efferent projection to the spinal cord. **(B)** Somatosensory neurons residing in the trigeminal send processes that innervate peripheral targets through the face, mouth, and dura and central targets in the brainstem. **(C)** Somatosensory neurons can be divided into three broad categories based on the size of their cell bodies and degree of myelination. Within these broad categories, numerous sub-specializations exist-for example small diameter C fibers are mostly nociceptors while large diameter A neurons respond to low threshold mechanical stimuli. LTMR, low-threshold mechano-receptor.

The cell bodies of the somatosensory neurons reside in ganglia that are found just outside the spinal cord (dorsal root ganglia or DRGs; Figure 1A) and at the base of the skull (trigeminal ganglia or TGs; Figure 1B). The sensory endings of the TGs cover the head and face, and those from the DRGs the rest of the body. These neurons are “pseudo-unipolar” with no dendrite but, instead, a single axon that bifurcates, creating one branch projecting to the periphery and another branch projecting into the central nervous system (CNS) at the level of the spinal cord and brainstem (Kandel et al., 2012; Purves et al., 2012).

Traditionally, somatosensory neurons were distinguished from one another through quantification of cell body size, degree of myelination, types of afferent endings, and the laminar targets of their efferent terminals in the spinal cord (Figure 1C; Kandel et al., 2012). Differences in anatomical features were correlated with physiological signatures that include receptive tuning, electrical properties, and neurochemistry. Using such criteria, somatosensory neurons were classified in four general fiber types: C, Aδ, Aβ, and proprioceptors (summarized in Table 1), each sub-serving broadly different roles in sensation. While this classification scheme has proved useful for many decades, it fails to account for the extraordinary molecular and functional diversity of somatosensory neuron subtypes that has emerged in recent years, and which is the focus of this review. Nevertheless, these categories are worth introducing, as their use remains common.

**Table 1:** **Somatosensory cell type markers**.

**Marker**	**Sensory cell type**
Parvalbumin	Proprioceptors and Aβ fibers
CGRP	Peptidergic C fibers, sub-population of Aδ fibers
Substance P	Peptidergic C fibers
NF200	Myelinated Aδ fibers, Aβ fibers, and proprioceptors
IB4	Non-peptidergic C fibers
Trpv1	Small diameter C fibers (heat and pain)
Trpm8	Small diameter C fibers (cold and pain)
MrgprD	Small diameter C fibers (noxious mechanical, pain)
MrgprA3	Small diameter C fibers (itch)
MrgprB4	Small diameter C fibers (innocuous mechanical)
VGlut3	Non-peptidergic C fibers (innocuous mechanical, cooling)
TH	Non-peptidergic C fibers (innocuous mechanical, cooling)
TrkB	Aδ fibers (innocuous mechanical, cooling)
Npy2r	Aβ fibers (innocuous mechanical)
Chondrolectin	Aβ fibers (innocuous mechanical)
DOR	Sub-populations of non-peptidergic C fibers and myelinated NF200-positive fibers

*Abbreviations: CGRP, calcitonin gene-related peptide; NF200, neurofilament heavy chain 200; IB4, isolectin B4; Trpv1, transient receptor potential cation channel subfamily V member 1; Trpm8, transient receptor potential cation channel subfamily M member 8; MrgprD, Mas-related G-protein-coupled receptor D; MrgprA3, Mas-related G-protein-coupled receptor A3; MrgprB4, Mas-related G-protein-coupled receptor B4; VGluT3, vesicular glutamate transporter type 3; TH, tyrosine hydroxylase; TrkB, tyrosine receptor kinase B; Npy2r, neuropeptide Y receptor type 2; DOR, delta opioid receptor*.

C fibers are the most abundant type, making up more than half of all somatosensory neurons (Kandel et al., 2012; Purves et al., 2012). They have small cell bodies, ranging in diameter from 10 to 30 μm. They are unmyelinated and thus conduct slowly (~2 m/s). C fibers are capable of responding to differing combinations of temperatures, pruritogens, tissue damage, chemical irritants, and mechanical stimuli (Figure 1C). C fibers are often defined as falling within two broad categories, peptidgergic and non-peptidergic. These classes can be distinguished by the expression of neuropeptides such as substance P or calcitonin gene related-peptide (CGRP; the peptidergic class of C fibers) or, conversely, binding by a histological marker called the isolectin IB4 (the non-peptidergic class of C fibers; Averill et al., 1995). However, some overlap between these markers exists (see below) and it is important to note that neuropeptides are found in other fiber types (McCoy et al., 2013). Additionally, there are other non-pepdidergic C fibers not labeled by IB4 (Abraira and Ginty, 2013). Thus, such markers are only partially selective. Activation of C fibers often, but not always, results in the sensation of pain (i.e., nociceptors). However, there are other classes of C fibers that respond rather specifically to cold, itch, or pleasurable touch (Zotterman, 1939). In the periphery, C fibers terminate as free nerve endings in the skin, organs, and bone. Centrally, they project to the superficial layers of the spinal cord (dorsal horn), a region known to be critical for the first stage processing of noxious and thermal stimuli (laminae I–III; Kandel et al., 2012; Purves et al., 2012).

Aδ fibers have medium diameter cell bodies and are lightly myelinated, and thus faster conducting than C fibers (up to 30 m/s). Like C fibers, they are responsive to combinations of temperature, force, and irritants (Kandel et al., 2012; Purves et al., 2012). A subset of Aδ fibers has been shown to be sensitive to innocuous temperatures (i.e., Hensel and Iggo, 1971; Darian-Smith et al., 1973; Duclaux and Kenshalo, 1980). Given their faster conduction velocity, it is believed additional classes are specialized for detecting highly localized “fast pain” rather than the more spatially diffuse “slow” pain associated with C fiber nociceptors (Basbaum et al., 2009). Aδ fibers also sensitize to repeated stimulation and/or tissue injury, thus playing a key role in inflammatory pain (Basbaum et al., 2009). Like C fibers, Aδ fibers project to the dorsal horn. These target similar superficial laminae as well as deeper layers of the spinal cord (lamina V) where they initiate nocifensive reflexes, such as innate withdrawal from noxious stimuli (for example, a hot stove).

In terms of size, the largest somatosensory neurons are the Aβ fibers dedicated to the detection of low-threshold mechanical stimuli and the Aα fibers that respond to muscle twitch (also called proprioceptors). Both types of somatosensory neurons have cell bodies averaging >50 μm. They are heavily myelinated and have fast conduction velocities (in the range of 30–70 m/s). Aβ fibers are low-threshold mechanoreceptors (LTMRs) and different subclasses are tuned to specific types of mechanical stimuli such as touch, vibration, and hair deflection. The nerve endings associated with Aβ fibers, including Meissner's corpuscles, Pacinian corpuscles, Merkel cell endings, and the lanceolate nerve endings that enwrap hair follicles, each display a unique morphology that is likely to sub-serve the tuning specializations of their associated nerve fibers (Kandel et al., 2012; Purves et al., 2012). Proprioceptor endings are also quite specialized. These innervate muscle spindles or Golgi tendon organs and are uniquely adapted to the detection of muscle tension and contraction. Centrally, Aβ fibers project to the dorsal column nuclei in the brainstem with branches in laminae III–V of spinal cord while proprioceptors also project to these deeper laminae, notably to local motor-stretch reflex circuits.

Despite decades of physiological and anatomical studies, many questions remained unanswered. In particular, an understanding of the signal transduction events lagged behind that for other sensory systems, notably olfaction, taste, and vision. Breakthroughs began to happen in the 1990's as genes critical to the sensing of somatosensory stimuli began to be discovered. Molecules required for pain signaling, such as sodium channels, as well as the receptors for key noxious agents, allowed researchers to address the specificity question from a new angle (Akopian et al., 1996; Caterina et al., 1997). These studies have helped elucidate the specificity of sensory neuron subtypes (i.e., Abrahamsen et al., 2008). Together with classical physiological studies, genetic studies are helping to create a clearer picture of how specific modalities are encoded by the somatosensory system.

## Transient receptor potential (TRP) channel expression differentiates somatosensory neuron subtypes dedicated to nociception and temperature detection

**Figure 2:**
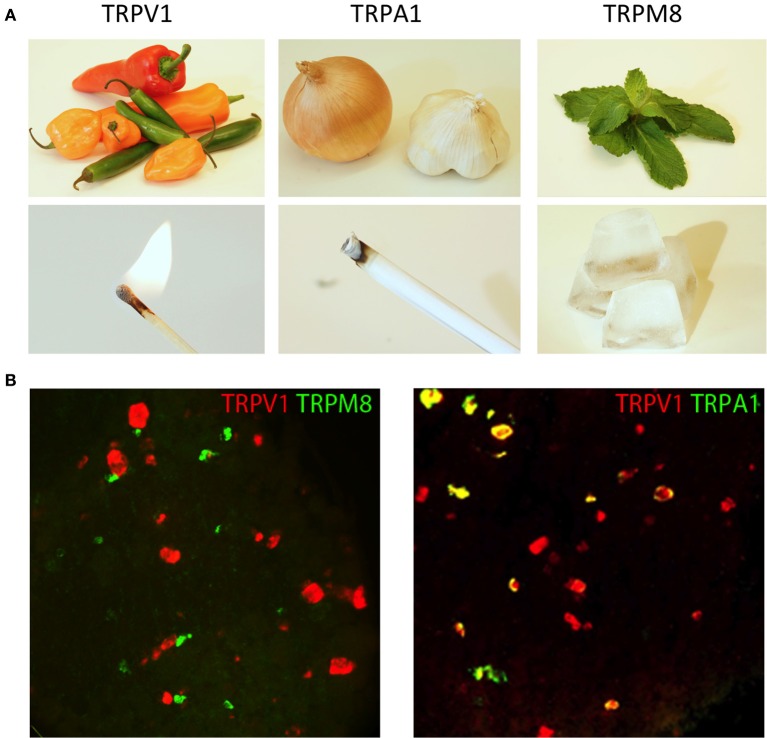
**TRP channels respond to unique types of stimuli. (A)** Natural products from chili peppers, onions/garlic, and mint leaves selectively activate TRP channels (TRPV1, TRPA1, and TRPM8, respectively). The psychophysical effects of these compounds directly correlate with the environmental stimuli to which these TRP channels are responsive. Activation of TRPV1 by either capsaicin from chili peppers or heat evokes a burning sensation, activation of TRPA1 by mustard oil and environmental irritants evokes a stinging pain, and activation of TRPM8 by menthol from mint leaves or cold evokes the sensation of cooling. **(B)** TRP channels are differentially expressed in somatosensory neurons. Two-color fluorescent *in situ* hybridization demonstrates little overlap between TRPM8 and TRPV1 (left hand image). Meanwhile, TRPA1 is expressed in a subset of TRPV1 neurons (right hand image). Image courtesy of Mark Hoon, NIH/NIDCD.

Work over the past two decades has uncovered a family of ionotropic receptors that respond to specific somatosensory stimuli (Figure 2A; Jordt et al., 2003; Bandell et al., 2007). Perhaps the most studied of these molecules is the non-selective cation channel, TRPV1 (transient receptor potential cation channel subfamily V member 1). Initially identified as the capsaicin receptor, TRPV1 is a principle detector of noxious heat (>42°C; Figure 2A; Caterina et al., 1997). TRPV1-knockout mice exhibit deficits in heat detection as measured by behavioral assays (Caterina et al., 2000). It is notable that the behavioral responses to heat are only diminished, suggesting that other sensory pathways for noxious temperature detection must exist (more on this below). TRPV1 is also activated by tissue acidification and lipid metabolites (Tominaga et al., 1998). The receptor becomes sensitized by many of the intracellular signaling pathways that are engaged during injury and inflammation (Chuang et al., 2001). Corroborating such findings, TRPV1-knockout mice have been shown to develop less thermal hyperalgesia in the context of inflammation, lending credence to the notion that TRPV1 is critical for noxious heat detection (Caterina et al., 2000). These studies have led to the emergence of TRPV1 as a therapeutic target of numerous pharmaceutical companies for pain management (Patapoutian et al., 2009).

In a similar manner, other TRP channels are expressed in unique subpopulations of sensory neurons. They too are tuned to unique forms of somatosensory stimuli (Figure 2B). For example, TRPA1 (transient receptor potential subfamily A member 1) is expressed in a subset of TRPV1^+^ neurons where it can be directly activated by pain-inducing chemical irritants, such as mustard oil, or indirectly activated through cellular signaling, such as calcium or GPCR activation (Jordt et al., 2004; Bautista et al., 2006; Kwan et al., 2006). In contrast, TRPM8 (transient receptor potential cation channel subfamily M member 8) is expressed in a largely non-overlapping subset of small-diameter neurons mostly lacking both TRPV1 and TRPA1. TRPM8 is activated by cooling (<27°C) as well as natural products that induce the sensation of cooling such as menthol (McKemy et al., 2002; Peier et al., 2002). Remarkably, mice in which TRPM8 is genetically deleted have profound defects in detecting cold temperatures (Bautista et al., 2007; Colburn et al., 2007; Dhaka et al., 2007).

Given the properties of these channels and the consequences of perturbing their function, it is believed that TRPV1 is a marker for nociceptors tuned to heat. A subset of TRPV1 neurons co-expressing TRPA1 is thought to detect chemical irritants and pruritogens. The TRPM8^+^ neurons, in contrast, are a relatively rare population of neurons that are thought to be cold sensors. Recently, several lines of mice have been generated to label and manipulate these neurons *in vivo* to directly test the properties of these neurons.

### TRPV1 reporter mice reveal the anatomy of a class of nociceptors

**Figure 3:**
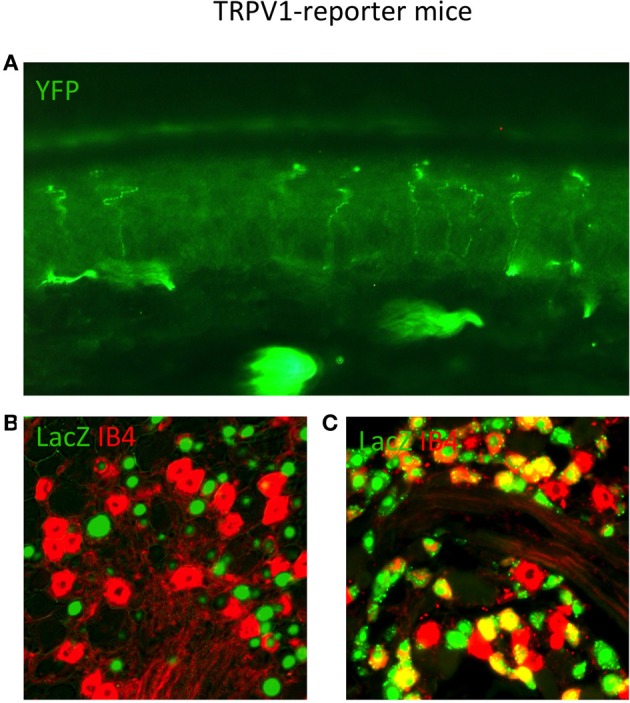
**TRPV1 Reporter mice reveal the anatomy of neurons that expressed TRPV1 throughout their lineage. (A)** Skin section from TRPV1-Cre × YFP reporter strain showing primary afferent arborizations in the various epidermal layers. **(B)** DRG section from adult TRPV1-PLAP-nlacZ mouse, showing minimal overlap between β-Gal reaction product (green) and IB4 (red). **(C)** DRG section from adult TRPV1-Cre × LacZ reporter strain. Anti-LacZ (green) shows significantly more overlap with IB4 (red) since the entire TRPV1 lineage is marked. Image courtesy of Danial Cavanaugh and Allan Basbaum, Department of Anatomy UCSF.

Upon the discovery of TRPV1, several anatomical methods were used to examine the expression pattern of this important receptor (Caterina et al., 1997). *In situ* hybridization offered excellent sensitivity but lacked the ability to determine neuronal morphology. Antibodies to TRPV1 were developed, but these had limited sensitivity and specificity. Thus, a more elegant solution was to capitalize on the power of mouse genetics and engineer a strain that selectively expressed a highly sensitive marker in all TRPV1^+^ neurons (Cavanaugh et al., 2011b). To examine the expression of TRPV1 *in vivo*, a mouse was created in which the placental alkaline phosphatase (PLAP) and a nuclear-localized β-galactosidase gene (nLacZ) were knocked in just after the stop codon of TRPV1 (Figure 3; Table 2; Shah et al., 2004). Internal ribosomal entry sites (IRES) were used such that TRPV1, PLAP, and nLacZ were translated as three independent proteins. Thus, the function of TRPV1 protein was preserved while allowing for the co-expression of two independent histological markers from the same mRNA transcript. Critically, this strategy preserved all the genomic regulatory elements upstream of the TRPV1 start site, ensuring the most faithful expression of the markers as possible.

**Table 2:** **Summary of the mouse lines discussed, with nerve ending descriptions for the neuron types labeled**.

**Mouse line #**	**Mouse line**	**Sensory cell type labeled or ablated**	**Peripheral nerve ending (skin)**	**Central projection (spinal cord)**	**Original reference(s)**
1	TRPV1-i-PLAP-i-nLacZ	Small diameter C fibers and subset of medium diameter Aδ fibers (heat, pain)	Free nerve endings	Laminae I–II and V	Cavanaugh et al., 2011a
2	TRPV1-Cre	Small diameter C fibers and subset of medium diameter Aδ fibers (heat, pain)	Free nerve endings	Laminae I–II and V	Cavanaugh et al., 2011b; Mishra et al., 2011
3	TRPV1-DTR	Small diameter C fibers and subset of medium diameter Aδ fibers (heat, pain)	Free nerve endings	Laminae I–II and V	Pogorzala et al., 2013
4	TRPM8-GFP	Small diameter C fibers and subset of medium diameter Aδ fibers (cold, pain)	Free nerve endings	Primarily lamina I	Takashima et al., 2007
5	TRPM8-fGFP	Small diameter C fibers and subset of medium diameter Aδ fibers (cold, pain)	Free nerve endings	Primarily lamina I	Dhaka et al., 2008
6	TRPM8-DTR	Small diameter C fibers and subset of medium diameter Aδ fibers (cold, pain)	Free nerve endings	Primarily lamina I	Pogorzala et al., 2013
7	CGRP-GFP-DTR	Peptidergic small diameter C fibers and subset of medium diameter Aδ fibers (heat, pain)	Free nerve endings	Laminae I–II and V	McCoy et al., 2013
8	MrgprD-EGFP	Small diameter C fibers (high threshold mechanical, pain)	Free nerve endings	Lamina II	Zylka et al., 2005
9	MrgprD-PLAP	Small diameter C fibers (high threshold mechanical, pain)	Free nerve endings	Lamina II	Zylka et al., 2005
10	MrgprD-DTR	Small diameter C fibers (high threshold mechanical, pain)	Free nerve endings	Lamina II	Cavanaugh et al., 2009
11	MrgprD-ChR2	Small diameter C fibers (high threshold mechanical, pain)	Free nerve endings	Lamina II	Wang and Zylka, 2009
12	Nav1.8:ChR2	Small diameter C fibers (all types)	Free nerve endings	Laminae I–II and V	Madisen et al., 2012; Daou et al., 2013
13	MrgprA3-EGFP:Cre	Non-peptidergic, small diameter C fibers (itch)	Free nerve endings	Lamina II	Han et al., 2012
14	MrgprB4-PLAP	Non-peptidergic, small diameter C fibers (stroking)	Free nerve endings	Lamina II	Liu et al., 2007
15	MrgprB4-tdTomato-2A-Cre	Non-peptidergic, small diameter C fibers (stroking)	Free nerve endings	Lamina II	Vrontou et al., 2013
16	VGLUT3-EGFP	Non-peptidergic, small diameter C fibers (low threshold mechanical)	Longitudinal lanceolate endings	Lamina II	Seal et al., 2009
17	VGLUT3-KO	Non-peptidergic, small diameter C fibers (low threshold mechanical)	Longitudinal lanceolate endings	Lamina II	Seal et al., 2009
18	TH-CreER	Non-peptidergic, small diameter C fibers (low threshold mechanical)	Longitudinal lanceolate endings	Lamina II	Li et al., 2011
19	TrkB-tauEGFP	Medium diameter Aδ fibers (low threshold mechanical)	Longitudinal lanceolate endings	Laminae II–III	Li et al., 2011
20	Npy2r-GFP	large diameter Aβ fibers (low threshold mechanical)	Longitudinal lanceolate endings	Laminae III–V	Li et al., 2011
21	Chondrolectin-PLAP	Large diameter Aβ fibers (low threshold mechanical)	Longitudinal lanceolate endings (in mystacial pad)	No data	Sakurai et al., 2013
22	Parvalbumin:Cre	Large diameter Aα and Aβ fibers (low threshold mechanical; and muscle tension and contraction)	Muscle spindle, Golgi tendon; Merkel and lanceolate endings (in mystacial pad)	Laminae III–V	Sakurai et al., 2013
23	DOR-EGFP	Sub-populations of both non-peptidergic C fibers and myelinated NF200-positive fibers (mechanical pain)	Meissner corpuscles, Merkel, and circumferential endings	Laminae I–V	Scherrer et al., 2009; Bardoni et al., 2014

**Figure 4:**
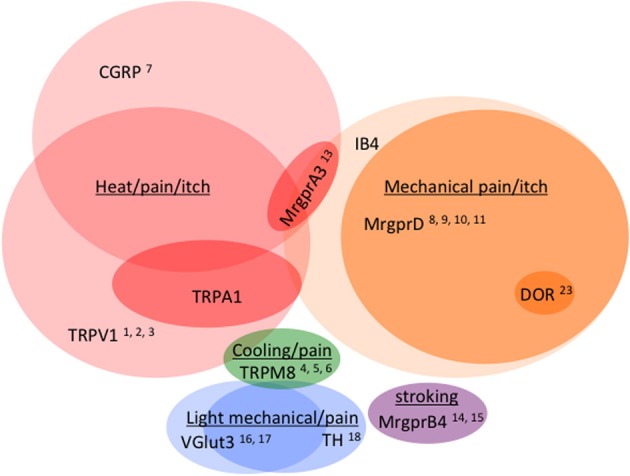
**Venn diagram illustrating the distribution of markers across different classes of C fibers (not to scale)**. Numbers reference the mouse lines listed in Table 2.

The numbers in the first column are referred to in Figure 4 to show the origin of the data classifying the neuron types. Abbreviations: TRPV1, transient receptor potential cation channel subfamily V member 1; i, IRES; PLAP, placental alkaline phosphatase; nLacZ, nuclear-localized β-galactosidase; Cre, cre recombinase; DTR, diphtheria toxin receptor; TRPM8, transient receptor potential cation channel subfamily M member 8; GFP, green fluorescent protein; fGFP, farnesylated green fluorescent protein; CGRP, calcitonin gene-related peptide; MrgprD, Mas-related G-protein-coupled receptor D; ChR2, channelrhodopsin 2; Nav1.8, voltage-gated sodium channel 1.8; MrgprA3, Mas-related G-protein-coupled receptor A3; MrgprB4, Mas-related G-protein-coupled receptor B4; VGluT3, vesicular glutamate transporter type 3; TH, tyrosine hydroxylase; TrkB, tyrosine receptor kinase B; Npy2r, neuropeptide Y receptor type 2; DOR, delta opioid receptor.

As observed by more traditional neuroanatomical techniques, nLacZ and PLAP staining in the adult reporter mice indicated that TRPV1 expression is enriched in peptidergic C fibers (Cavanaugh et al., 2011a). The TRPV1^+^ neurons accounted for nearly all of the unmyelinated, peptidergic DRG neurons while being largely absent from the non-peptidergic C fibers as defined by IB4 (Figure 4). Highlighting the utilities of the reporter expression technique, the greater sensitivity of nLacZ staining revealed neurons with weak TRPV1 expression that were previously missed by standard approaches relying on TRPV1-specific probes and antibodies. The increased sensitivity resulted in the unexpected observation that a significant number (>20%) of TRPV1^+^ neurons are negative for both peptidergic and non-peptidergic markers. Thus, the traditional classes of peptidergic and IB4-binding non-peptidergic C fibers do not account for the entirety of small diameter neurons (more on this below).

The improved sensitivity of PLAP staining also afforded a much more detailed picture of the peripheral and central projections of TRPV1^+^ sensory neurons with single fiber resolution (Cavanaugh et al., 2011a,b). Staining revealed extensive axonal labeling of peripheral tissues in numerous areas, including the bladder, cornea, and glabrous and hairy skin of the hind paw, as well as previously unobserved single axonal endings that terminated in the outermost epidermal layers. The analysis of these mice has also led to a greater appreciation of the extent of primary afferent innervation in central tissues. PLAP staining revealed numerous projections from trigeminal and nodose ganglia to regions in the brainstem and medulla such as the superficial layers of the medullary dorsal horn (also referred to as the nucleus caudalis), the nucleus of the solitary tract, and the area postrema. PLAP^+^ fibers found in the region of the rostral ventrolateral reticular nucleus raised the intriguing possibility of a monosynaptic input from TRPV1^+^ afferents to motor neurons of the nucleus ambiguus. Additionally, axons were observed coursing throughout the parabrachial nucleus suggesting significant monosynaptic input from nociceptors (Cavanaugh et al., 2011b). This is of particular interest since parabrachial neurons in turn project to the amygdala, which has been implicated in emotional aspects of the pain experience (Feil and Herbert, 1995; Jasmin et al., 1997).

In addition to its well-established role in somatosensation, a flurry of recent papers suggests TRPV1 might be functioning in other tissues (Kauer and Gibson, 2009). Particular focus has been in the CNS, where TRPV1 has been proposed to participate in a myriad of functions that include osmodetection in the hypothalamus and/or the modulation of synaptic strength in several brain regions (Sharif Naeini et al., 2006; Chávez et al., 2010; Grueter et al., 2010). Given the exquisite sensitivity of the reporter strain, one would expect to find significant PLAP and/or nLacZ staining within the brains of the reporter mice (Cavanaugh et al., 2011a). Given nLacZ's nuclear localization coupled with the enzymatic amplification of the X-gal reaction used for detection, this is likely the most sensitive means of detecting TRPV1 expression to date. Quite surprisingly, such analyses failed to detect TRPV1 expression in the populations of neurons involved in the previously described forms of TRPV1-dependent synaptic plasticity. That said, a scattering of cells positive for nLacZ was observed within the CNS. This analysis, coupled with RT-PCR and radioactive *in situ* hybridization showed that TRPV1 is expressed in neurons that are present in a limited number of brain regions, the most prominent of which are in a contiguous band, centered along the midline of the posterior hypothalamus and rostral midbrain (Cavanaugh et al., 2011b).

In light of the sensitivity of the detection method, the absence of CNS staining almost certainly reflects a level of TRPV1 expression far below anything functionally meaningful. It seems very unlikely that the physiological and/or pharmacological effects reported could be mediated by TRPV1 as it would require these processes to be working on only a handful of molecules that the cell might be expressing. That said, there does seem to be appreciable expression of TRPV1 in a discrete population of neurons in the posterior hypothalamus, although its functional consequence remains to be determined.

### Examining the lineage of TRPV1-expressing neurons with Cre-reporter mice

Two groups constructed mice where Cre recombinase is expressed under control of the TRPV1 promoter either via direct knock-in to the TRPV1 locus (Cavanaugh et al., 2011a,b) or by using transgenics based on Bacterial Artificial Chromosomes (BACs; Mishra et al., 2011). Both groups crossed the resultant mice strains to ROSA-reporter strains. Since Cre-mediated genetic recombination is irreversible, crossing the TRPV1-Cre mice to floxed reporter strains allows one to mark all the cells that expressed TRPV1 throughout development, even transiently. Indeed, when these crosses were generated, >50% more labeled neurons were observed as compared to the TRPV1-PLAP-nLacZ mice. This expansion of labeled cells reflects the much broader expression of TRPV1 during development and its eventual down-regulation after birth. Also of note, neither group observed appreciable numbers of labeled neurons in the CNS with this highly sensitive approach, further supporting the conclusion that TRPV1 is unlikely to be expressed widely in the brain, even during embryonic development. Sensory neurons that transiently expressed TRPV1 included neurons dedicated to cold temperatures, as evidenced by the co-expression of the marker with TRPM8.

### Visualizing cold-sensing neurons with TRPM8 reporter mice

Two distinct populations of cold-sensing neurons have been identified in human and primates—slowly conducting neurons that are believed to signal cold pain (>18°C) and faster conducting neurons believed to be Aδ fibers that respond to innocuous cooling (Darian-Smith et al., 1973; Dubner et al., 1975; Georgopoulos, 1976). Just as TRPV1 expression has been successfully exploited to label heat-sensitive somatosensory neurons, the expression of the menthol/cold receptor TRPM8 has been used to delineate those neurons believed to be dedicated to the detection of cold. Given that the population of TRPM8-expressing neurons is relatively small (~10% of DRG neurons), transgenic labeling provides an excellent means of locating these neurons in tissue sections, *in vitro*, and *in vivo*. Two lines of mice have been generated that express variants of GFP under the control of the TRPM8 promoter. Interestingly, while these mice have many similarities, their construction methods and analyses have some notable differences.

Takashima et al. used a BAC-transgenic approach to express GFP in TRPM8^+^ neurons while leaving the endogenous promoter region untouched (Takashima et al., 2007). The BAC approach has the added benefit that TRPM8 protein is also expressed normally and the mice retain normal behavior responses to cooling. The large genomic regions included in BACs are preferable to smaller transgenes in that they often retain the long-range regulatory elements required for faithful gene expression and, as such, these types of transgenic mice suffer less from positional effects and/or multiple random targeting events (Heintz, 2001). However, as with small transgenes, careful analyses are still required to ensure the expression observed truly reflects endogenous patterns.

In order to validate the TRPM8 reporter strain, several key predictions from the Takashima study needed to be examined. For example, it is well established that TRPM8 is robustly activated by menthol, a cooling compound found in mint leaves, and the receptor is the sole detector of this molecule in sensory neurons (Bautista et al., 2007; Dhaka et al., 2007). Therefore, one would expect that the preponderance of GFP^+^ neurons in the BAC-transgenic TRPM8-GFP mouse line should be sensitive to this compound. Analyses of these mice found that 81.5 ± 4.8% of the GFP^+^ DRG neurons were menthol-sensitive. While this is an impressive degree of overlap, it is important to consider the 18.5% of neurons that failed to respond. It is difficult to determine from this study if these neurons were not expressing TRPM8 at sufficient levels, were physiologically compromised, or, more importantly, whether GFP expression was the result of some “leaky” transcription from the BAC promoter.

A separate group used an alternate strategy to create a TRPM8 reporter mouse. Rather than using BAC transgenesis, Dhaka et al. (2008) generated mice via gene targeting in which the TRPM8 coding sequence was replaced with a farnesylated GFP (fGFP), resulting in functional disruption of the receptor (Dhaka et al., 2008). However, since TRPM8 is haplosufficient, heterozygous mice retained normal responses to cooling and thus could be used for functional analyses. An interesting side note was that when these mice were first described in 2007, the authors originally failed to observe any fGFP expression (Dhaka et al., 2007). The marker expression was only recovered after the removal of loxP-flanked EM7-PGK-NEO cassette. Again, such nuances call attention to the delicate nature of genome engineering.

When Dhaka et al. subjected their strain to the same control testing as Takashima et al., their knock-in mice appeared to faithfully mark neurons expressing TRPM8. Dhaka et al. reported that 19/20 fGFP^+^ neurons responded to both cooling and menthol. The histochemical analyses from the two groups seemed to follow the same trend, with the BAC line expressing GFP in a greater percentage of DRG neurons than the knock-in line. One explanation for the discrepancy is that the BAC line captures even extremely low levels of TRPM8 expression, below what would be required for functional detection. Therefore, the knock-in mouse seems to more conservatively estimate the true TRPM8 population while perhaps missing some cells with low-level expression while the BAC line may be overestimating the extent of TRPM8 expression.

Regardless of the differences, just as with the TRPV1 reporter mice, both TRPM8 strains provided far more sensitive detection than previously available. While TRPM8 *in situ* probes work beautifully, the generation of effective antibodies has proven more challenging. Therefore, these new strains offer the opportunity to better resolve the neurochemistry of TRPM8^+^ neurons through co-labeling with antibodies as well as the chance to observe the projection patterns of TRPM8^+^ afferents at single fiber resolution. Both studies conclude that TRPM8 is expressed predominantly in both C and Aδ fibers that completely lack binding of the isolectin IB4 (Figure 4; Table 2). As reported previously, TRPM8^+^ neurons are found in greater percentages in the TG vs. the DRG (McKemy et al., 2002).

The analyses of these two strains reveal, as with TRPV1, that TRPM8^+^ neurons are not homogenous but in fact are likely comprised of distinct subtypes dedicated to unique aspects of cold sensation. For example, while the numbers in the two studies differ, it is clear that a significant proportion of TRPM8 neurons co-express TRPV1 (18% of TG neurons in the knock-in vs. 38.8% in the BAC). It is tempting to speculate that these neurons are nociceptors that report on painful temperatures regardless of whether they are cold or hot. Moreover, given that both TRP channels are modulated by intracellular signaling pathways that are engaged during injury and inflammation, these neurons are strong candidates for being critical actuators of hyperalgesia at both ranges of temperatures. A distinct population of TRPM8^+^ neurons lacked all known markers for nociceptors entirely, suggesting these are a unique group of cells. One possibility is that these neurons encode the sensation of cooling (rather than cold pain). Finally, Takashima et al. present evidence that a sub-population of Aδ fibers are TRPM8^+^, thereby identifying another unique set of neurons that possibly report on either innocuous cooling or perhaps the fast component of cold pain. Indeed, physiological studies point to such neurons existing in humans (Hensel and Iggo, 1971).

A great benefit of reporter mouse strains is the ability to visualize projections with up to single fiber resolution and these studies, for the first time, provide a map of the nerve endings of cold sensors throughout the body. Analyses from these strains revealed that TRPM8^+^ free nerve endings could be found in multiple layers of the epidermis of glabrous skin (stratum granulosum and stratum spinosum) while they were mostly absent from the dermis. These fibers also target the epidermis of the intervibrissal fur of the mystacial pad, fungiform papillae of the tongue, and both the dentin and pulp of teeth. The latter is one of particular interest given the well-documented sensitivity of teeth to cold stimuli.

The central projections of TRPM8^+^ neurons target the dorsal spinal cord. As demonstrated with antibody staining, TRPM8^+^ fibers largely terminate in the most superficial layer (lamina I; Bautista et al., 2007). This layer receives most of the input from peptidergic nociceptors with no projections to lamina II (as evidenced by the lack of overlap with IB4^+^ projections or PKCγ^+^ spinal interneurons) or lamina III. Therefore, it appears that cooling information is specifically routed to the most superficial layers of the spinal cord.

Finally, Dhaka et al. observed a small cohort of GFP^+^ neurons (22%) from homozygous mice (hence completely deficient in TRPM8 expression) that retained responses to cold, albeit at temperatures far below the threshold reported for TRPM8, thus suggesting an alternative cold detection pathway may exist in these neurons. Physiological responses to temperatures below 0°C in nerve recordings in rats have been demonstrated to be more widespread than those reported for cloned TRPM8 channels (<18°C; Simone and Kajander, 1997). This is unlikely to be attributable to TRPA1. TRPA1 and TRPM8 are not co-expressed and despite early reports, it seems unlikely that TRPA1 contributes to cold sensation in mice (Bautista et al., 2006). Indeed, such a finding is consistent with studies showing that TRPM8-knockout mice retain behavioral responses to extreme cold that persist even in TRPM8/TRPA1 double knockouts (Knowlton et al., 2010).

### Cell type-specific ablation of TRP channel expressing neurons define the roles of specific sensory neuron classes in temperature sensation and pain

In addition to marking sub-populations for anatomical studies, TRP channel loci have also been exploited to manipulate the function of heat/cold sensing neurons. Mishra et al. crossed ROSA-stop-DTA mice, which conditionally express the diptheria toxin (DTA), to TRPV1-Cre strains, to generate offspring in which any neuron in the TRPV1 lineage would selectively undergo cell death (Mishra et al., 2011). Since TRPV1 is more widely expressed in somatosensory neurons during development, these mice lack neurons expressing TRPV1, TRPA1, and TRPM8 (Cavanaugh et al., 2011a; Mishra et al., 2011). Thus, offspring were found to have profound deficits in the detection of a wide range of temperatures (−5 to 55°C), chemical irritants, and pruritogens. More importantly, the mice retained the ability to respond to light touch and mechanical pain, underscoring the selectivity of the ablation strategy. Also worth noting, in other behavioral tests (such as for cognition and locomotion) these mice were completely normal, again arguing against widespread TRPV1 expression in the CNS.

Since several classes of sensory neurons were ablated in TRPV1-Cre X ROSA-stop-DTA mice, alternate strategies were needed to selectively ablate the neurons that express TRPV1 in adult mice. While several groups have used strong TRPV1 agonists to induce excitotoxicity, a more refined genetic approach is preferable, since it does not rely on strong overstimulation of neural circuits or incomplete ablation (Mishra and Hoon, 2010). Using BAC transgenics, Pogorzala et al. (2013) addressed this need by creating a new strain of mice that express the diptheria toxin receptor (DTR) directly from the TRPV1 promoter via an expression cassette that also included a separately translatable GFP-coding region, to allow for direct observation of the neurons (Pogorzala et al., 2013). Expression of DTR renders neurons susceptible to ablation through systemic injection of diphtheria toxin.

Diptheria toxin administration to adult TRPV1-DTR mice resulted in complete ablation of all TRPV1-expressing neurons. After ablation, these mice retained normal responses to cold but completely lacked aversion to noxious heat (40–50°C). Therefore, TRPV1 expression in the adult defines the sensory neurons needed for encoding heat stimuli. The phenotype created by ablating these neurons was much more profound compared to genetic deletion of TRPV1. Thus, there must be additional pathways through which heat stimuli activate these cells. As an alternative to the existence of specific receptors for extreme heat, it is possible that factors released after cellular damage (such as ATP and protons) might stimulate non-heat sensitive receptors, such as P2X purinergic receptors and acid-sensing ion channels (ASIC; Birdsong et al., 2010). That said, other ion channels expressed in somatosensory neurons have been demonstrated to be responsive to thermal stimuli (Vriens et al., 2011).

It is also remarkable that, despite their lack of responses to noxious heat, mice with all their TRPV1-expressing neurons ablated retained normal responses to cold. Therefore, the detection of different temperatures is encoded by separately dedicated thermosensitive neurons. As discussed above, TRPM8 expression seems to completely describe the cold-sensing neurons. To formally test this hypothesis, transgenic mice were generated to selectively ablate this sub-population of sensory neurons (Pogorzala et al., 2013). Analysis of the TRPM8-DTR mouse revealed that these neurons are required for sensing cold. Much like the TRPV1-DTR mice, the phenotype was more severe than the genetic deletion of the receptor alone, with deficits in the detection range down to 0°C.

TRPM8-DTR mice had normal responses to heat, thus reinforcing the notion of distinct channels for hot and cold sensation. Indeed, when the TRPM8-DTR mice were crossed to TRPV1-DTR mice, the resultant depletion of both populations could roughly be approximated by the addition of the two phenotypes. Even given the removal of the hot- and cold-dedicated neurons, there remained some responses to extreme temperatures. Such responses may be due to molecules (such as lipids and/or ATP) released during tissue damage and sensed by other classes of nociceptors (see below).

Finally, by removing the hot and cold channels, these mice no longer were attracted to warm. Thus, one possible conclusion is that all the information needed to detect the complete range of physiologically relevant temperatures is accounted for by TRPV1- and TRPM8-expressing somatosensory neurons. In the simplest model, attraction to warmth would be viewed as the balance between two aversive stimuli, in this case hot and cold. However, it is important to point out that an alternative hypothesis is equally supported by the current data. One cannot rule out the possibility that there exist subsets of neurons whose specific activation by warm temperatures produce attraction. Physiological studies in human and primates from the 1970s support this latter model (i.e., Hensel and Iggo, 1971).

## Defining a role for the peptidergic class of sensory neurons

Since CGRP (calcitonin gene-related peptide) has been traditionally used as a marker of peptidergic C fibers, what would be the phenotype of a mouse lacking CGRP^+^ peptidergic fibers? Based on the coexpression of TRPV1 in some but not all of these neurons, one might have predicted they would lack some type of heat sensation. To study this subset of peptidergic C fibers, McCoy et al. (2013) generated a mouse in which the peptidergic CGRPα-expressing cells could be visualized by means of expression of a fGFP knocked into the CGRPα locus (CGRPα-GFP). The construct also included a loxP-STOP-DTR sequence, enabling the recombination of diphtheria toxin receptor (DTR) into the CGRPα locus in Cre-expressing cells (CGRPα-GFP-loxP-STOP-hDTR mice; Table 2) and thus the ability to selectively ablate these neurons. When crossed with an advillin-Cre line that expresses Cre in sensory neurons only (Minett et al., 2012), the CGRPα-positive DRG neurons are sensitive to ablation by administration of diphtheria toxin (DTX). The advillin promoter provided sensory neuron specificity, leaving other central CGRP-expressing cells intact.

Using quantification of immunostained sections, McCoy et al. (2013) show that in DTX-treated mice, >90% CGRP^+^ DRGs are ablated, and this includes 50% of TRPV1^+^ cells as well as 36% of IB4-binding cells. TRPM8^+^ cells, on the other hand, were spared. In concordance with these findings, through electrophysiological recordings in an *ex vivo* skin-nerve preparation in which an intact nerve still attached to skin is stimulated in the periphery while isolated fibers are recorded from, the ablated mice were shown to lack sensitivity to noxious heat but not to cold or mechanical stimulation. In behavior tests, the ablated mice showed a loss of heat sensitivity (shown in 3 tests: tail immersion at 46.5 and 49°C, hot plate at 52°C, and intraplantar capsaicin injection) and no change to mechanosensitivity (von Frey, tail clip test for noxious mechanical, and cotton swab test for innocuous mechanical sensitivity). But surprisingly, they also showed an increase in cold sensitivity in various tests (acetone application to paw skin, tail immersion at −10°C, intraplantar injection of icilin, and cold plantar assay), and an enhanced avoidance of cooler temperatures in a two-choice test, in which the mouse can choose which of two plates (each set at a different temperature) to spend time on. The disparity between the electrophysiological and behavioral results suggested that the increase in cold sensitivity might be a central mechanism since the cold afferents appear to be normal in the ablated mice.

In a deeper examination of the intriguing finding that DTX-treated mice display behavioral changes to cold responses but no alteration in the electrophysiological response of their primary afferents to cold, the authors performed patch recordings from lamina II in spinal cord slices from these mice. Compared to saline-treated mice, the DTX-treated mice were less responsive to capsaicin, but more responsive to icilin both for number and frequency of events, and corresponding to a five-fold increase in tonic and evoked activity. The authors therefore hypothesized that the CGRPα^+^ DRGs normally provide tonic inhibition of TRPM8 neurons and thus when ablated, the TRPM8 input to the spinal cord is greater, causing hypersensitivity to cold. However, ablation of TRPV1 neurons does not result in cold hypersensitivity, thus suggesting this circuit may involve TRPV1^−^/CGRP^+^ neurons or that these ablations result in unexpected perturbations yet to be understood.

## Mas-related G-protein-coupled receptors mark distinct subtypes of C fibers dedicated to irritants and mechanosensation

While the TRPV1 and TRPM8 loci are invaluable tools for genetically manipulating the sensory neurons dedicated to temperature sensation, to define additional subtypes of C fibers, let alone all the different classes of somatosensory neurons, other markers are required. For example, TRPV1 and TRPM8 are both largely absent from non-peptidergic C fibers (Pogorzala et al., 2013). Moreover, as summarized above, the neurons that express either TRP channel can themselves be subdivided even further. Some TRPV1^+^ neurons might be dedicated to heat while others are responsive to irritants (Bautista et al., 2006). Some TRPM8^+^ neurons might be dedicated to innocuous cooling while others to cold pain (McKemy, 2013).

To gain further genetic access to additional C fiber classes, the loci from the Mas-related G-protein-coupled receptors (Mrgprs) have proven particularly effective. The Mrgprs are a large family of receptors expressed in sensory neurons, with >50 members in mice (Dong et al., 2001). In DRGs, differential expression of members of the four Mrgpr subfamilies (designated MrgprA-D) can be used to define distinct somatosensory neuron subtypes. While the majority of Mrgprs remain “orphan receptors” (i.e., their endogenous ligands are still unknown), anatomical studies utilizing reporter mice and functional studies relying on knock-in and knock-out mice have been instrumental in demonstrating that the expression of these genes delineates classes of neurons that respond to both noxious (mechanical pain, itch, and tissue damage) and innocuous (stroking) stimuli.

### MrgprD marks non-peptidergic polymodal C fibers

Two knock-in reporter strains have been generated that express either EGFP or PLAP under control of a single Mrgpr gene by utilizing the endogenous MrgprD promoter (Zylka et al., 2005). Analysis revealed that MrgprD is expressed in the majority of non-peptidergic C fibers that bind the isolectin IB4 (>75%). Remarkably, MrgprD^+^ neurons exclusively innervate hairy and glabrous skin, where they overwhelmingly form free nerve endings throughout the epidermis. The fibers preferentially terminate in the *stratum granulosum* and make up ~60% of all free nerve endings present there. Histochemical analysis of these mice revealed that peptidergic and non-peptidergic fibers are uniquely distributed in skin, further supporting the model that somatosensory neurons form highly specialized subpopulations.

Similar to the exquisite specificity observed in peripheral innervation, MrgprD^+^ projections form a narrow band within lamina II (also called the *substantia gelatinosa*, or SG) of the dorsal horn of the spinal cord. Quite unexpectedly, the MrgprD^+^ projections sit nicely between markers for lamina I and II_outer_ (as defined by CGRP staining) and lamina II_inner_ (as defined by PKCγ expression), thus refining our understanding of the anatomical specialization that exists within a single lamina in the dorsal horn, in this case lamina II. Such specificity suggests the information coding in the dorsal spinal cord should exhibit a high degree of regional specialization. For example, based on MrgprD projection patterns, the middle of lamina II may be biased toward encoding nociceptive information generated from the outer layers of the epidermis of hairy and glabrous skin.

In much the same way as the roles of TRPV1^+^ and TRPM8^+^ neurons were assessed, the contributions of MrgprD^+^ neurons to behavior were tested using a genetic ablation strategy. MrgprD knock-in mice were generated where the coding region of MrgprD was replaced with a DTR-ires-EGFPf cassette (Cavanaugh et al., 2009). When MrgprD^+^ neurons were ablated in adult mice, there was a significant reduction in the responses to noxious mechanical stimuli. Mice lacking MrgprD^+^ neurons required larger forces from a von Frey filament than wild-type littermates to elicit a paw withdrawal. MrgprD-DTR mice also exhibited a reduction in mechanical hypersensitivity after inflammation induced by Complete Freund's adjuvant (CFA) following toxin administration. The deficits were modality-specific in that the responses of the MrgprD-DTR mice to thermal stimuli remained indistinguishable from wild-type mice. However, unlike the deletion of hot or cold neurons, which produced complete insensitivity to thermal stimuli, mice depleted of MrgprD^+^ neurons retained some ability to sense noxious mechanical stimuli, albeit with less sensitivity. This is not unexpected given the majority of somatosensory neurons have some ability to respond to mechanical stimulation (Delmas et al., 2011). That said, the remaining sensitivity to noxious mechanical stimuli cannot be attributed to TRPV1^+^ neurons. Silencing of heat-sensitive neurons in the MrgprD-ablated mice revealed no additional mechanosensory deficits. Therefore, other classes of neurons must contribute to sensing noxious mechanical stimuli.

While co-deleting both the MrgprD^+^ and TRPV1^+^ neurons in a single mouse does not enhance deficits in mechanosensation, it does impact the ability to detect painful temperatures. As mentioned above, while deletion of TRPV1^+^ sensory neurons clearly results in profound deficits, behavioral responses to temperatures >50°C remain. Such nocifensive responses are significantly blunted by removing both sub-populations of neurons such that co-deletion results in mice that will stand on a hot plate at 55°C as if it were at room temperature (Pogorzala et al., 2013). Notably, MrgprD^+^ neurons also appear to contribute to extreme cold sensation. In much the same manner, co-deleting MrgprD^+^ and TRPM8^+^ neurons results in mice with significantly less cold sensitivity than mice lacking TRPM8^+^ neurons alone. While one cannot rule out that at least some MrgprD^+^ neurons selectively respond to noxious hot and cold, it seems unlikely. The simplest explanation is extreme temperatures result in factors being released from damaged tissue and it is these molecules that stimulate MrgprD^+^ neurons. Indeed, MrgprD neurons have been shown to be robustly activated by extracellular ATP (Dussor et al., 2008).

Finally, pharmacological activation of MrgprD^+^ neurons causes a mild itch response. Injection of β-alanine, a MrgprD ligand, into mouse cheeks provokes a scratching behavior that require MrgprD^+^ neurons (Han et al., 2012; Liu et al., 2012). A high dose of β-alanine, which is used as a nutritional supplement for body builders, evokes paresthesia in humans that include tingling, pins and needles, and numbness. Whether MrgprD^+^ neurons are homogenous or comprise multiple sub-populations remains to be seen.

### MrgprA3 defines a class of sensory neurons dedicated to itch

In addition to temperature and noxious mechanical stimuli, nociceptors are also required for the detection of pruritogens. Numerous compounds exist that can evoke scratching behavior, the most widely studied being histamine, which signals through TRPV1^+^ neurons (Shim et al., 2007; Imamachi et al., 2009). However, several itch conditions, such as atopic dermatitis, operate through non-histaminergic pathways (Wilson et al., 2013). One such pathway is the itch that is induced as a side effect of taking the anti-malarial drug chloroquine. Functional studies have found that MrgprA3 is the predominant chloroquine receptor expressed in sensory neurons in mice (Liu et al., 2009). MrgprA3 is both necessary and sufficient for the detection of chloroquine and activation of MrgprA3^+^ neurons results in robust scratching behavior.

To selectively label and identify sensory neurons dedicated to itch, a BAC transgenic mouse line was engineered that expresses a GFP-Cre fusion protein under control of the MrgprA3 promoter (Han et al., 2012). Thus, this approach enables transgene expression in itch-sensitive neurons as well as manipulation of gene expression within these neurons by means of Cre-mediated recombination. These mice were crossed to a Cre reporter line, yielding mice with a subset of sensory neurons that robustly expressed tdTomato, an exceptionally bright fluorescent protein that efficiently labeled both the cell bodies and fine processes of these neurons. The expression of tdTomato faithfully represented the distribution of the endogenous MrgprA3 gene product, as measured by overlaying the tdTomato fluorescence and *in situ* hybridization (>95% overlap). Critically, >90% of GFP^+^ neurons from these mice could be activated by chloroquine.

MrgprA3^+^ neurons represent a small percentage of DRG neurons (6.8%) and constitute a subpopulation of TRPV1^+^ neurons (19%). Co-labeling with a battery of markers revealed that the majority of MrgprA3^+^ neurons not only express TRPV1 (88.3%) but also the neuropeptide CGRP (84.2%). Interestingly, many also bind IB4 and thus make up a rare cohort of CGRP^+^ neurons that are also marked by the isolectin. Electrophysiological characterization of MrgprA3^+^ neurons revealed these are indeed polymodal nociceptors that respond not only to pruritogens but also noxious heat and mechanical stimuli.

Remarkably, the peripheral and central targets of MrgprA3^+^ neurons were identical to those of MrgprD^+^ neurons, even though these two receptors are not co-expressed in the same neurons. Both populations exclusively innervate the superficial epidermis of skin (*stratum granulosum*) and no other tissues in the body. They also both project to the middle of lamina II where their nerve endings are co-mingled. Given that activation of these two subpopulations of neurons evokes distinct sensations (mechanical pain vs. itch), mapping the circuitry of this region of the dorsal horn should prove to be fascinating. One dorsal horn cell type that is of particular interest are the gastrin-releasing peptide receptor (GRPR)^+^ neurons, known to be critical for the detection of pruritogens (Sun and Chen, 2007; Sun et al., 2009). Indeed, Han et al. provide evidence that links activation of MrgprA3^+^ cells to activity-dependent expression of the immediate early gene FOS in GRPR-neurons.

In the same vein as TRPV1, TRPM8, and MrgprD, conditional cell ablation was used to demonstrate the function of MrgprA3^+^ neurons *in vivo*. Crossing the MrgprA3-EGFP:Cre line with a ROSA-DTA strain allowed for selective ablation of virtually all MrgprA3^+^ neurons. While ablation had no effect on responses to noxious thermal and mechanical stimuli (cheek injection with capsaicin or allyl isothiocyanate, tail immersion test at 50°C, hot plate, and von Frey tests), these mice were compromised in their ability to respond to pruritogens. That said, scratching behavior persisted for almost all compounds tested and thus other neuronal populations must also contribute to the detection of pruritogens. These likely include additional TRPV1^+^ subpopulations, particularly those that co-express TRPA1 (Wilson et al., 2011).

While ablation strategies can be used to evaluate the contribution of a particular sub-population of sensory neurons to behavior, they do not speak to whether activation is sufficient. To address sufficiency, Han et al. generated a mouse strain that expresses the TRPV1 receptor solely in MrgprA3^+^ neurons. To accomplish this, MrgprA3-EGFP:Cre mice were crossed to a ROSA-TRPV1 strain, thus enabling robust expression of TRPV1 from the ROSA locus in all MrgprA3^+^ neurons (Arenkiel et al., 2008). When these mice were placed in a TRPV1-knockout background, the endogenous receptor was no longer expressed, leaving only MrgprA3^+^ neurons responsive to capsaicin. Normally, application of capsaicin elicits nocifensive behaviors. However, in the TRPV1 gain of function transgenic mice, capsaicin evoked scratching behavior instead. These experiments elegantly argue that activation of MrgA3^+^ neurons is sufficient to cause itch.

In addition to MrgprA3, another marker for sensory neurons dedicated to itch is TLR7 (Toll-like receptor 7). While reporter lines for TLR7 are still lacking there is some evidence that its expression is at least partially overlapping with MrgprA3 (Liu et al., 2010). The lack of sensitivity of TLR7 knockout mice to non-histaminergeic pruritogens demonstrates that TLR7 is a direct mediator of pruritis. TLR7 may also mediate itch through non-neuronal cells in the skin. Thus, transgenic strategies to label and manipulate these specific cell types should prove informative.

### MrgprB4 marks a unique class of C fibers that respond to gentle stroking

*In situ* hybridization studies have demonstrated that members of the Mrgpr family have non-overlapping expression patterns in small-diameter somatosensory neurons (Dong et al., 2001). Given the distinct functions of MrgprD^+^ and MprgprA3^+^ neurons, it stands to reason that other members of this receptor family can be used to distinguish additional subpopulations of C fibers. MrgprB4 is one member that marks a unique subset of small-diameter neurons that express neither MrgprD nor MrgprA3. To understand the anatomy of these neurons further, Liu et al. (2007) created a knock-in line that replaces the MrgprB4 coding region with PLAP. Analyses of these mice revealed that MrgprB4 marks an exceptionally small population of non-peptidergic, IB4^+^ small diameter somatosensory neurons (≤2%). Like MrgprD- and MrgprA3^+^ neurons, neurons expressing MrgprB4 specifically target the epidermis, but in this case show selectivity for hairy skin. MrgprB4^+^ free nerve endings show exceptionally large arborizations that are associated with hair follicles and the adjacent epidermis. Such arborization patterns resemble the C-tactile endings previously described in humans (Olausson et al., 2002). Centrally, MrgprB4^+^ fibers terminate in the same region of lamina II as MrgprD^−^ and MrgprA3^+^ neurons. Whether spinal horn neurons receive inputs from unique or multiple Mrgpr fiber types remains to be seen.

To investigate the functional specialization of the MrgprB4^+^ population, Vrontou et al. (2013) created a MrgprB4-tdTomato-2A-Cre mouse line. They first used this line to selectively express the genetically encoded calcium sensor GCamp3.0 in neurons expressing MrgprB4 (Tian et al., 2009). Expression was accomplished by infecting transgenic mice with a Cre-dependent adeno-associated virus (AAV). The authors developed an *in vivo* spinal cord preparation in which transduced MrgprB4^+^ fibers expressing GCaMP3 could be imaged by two-photon microscopy while being stimulated in the periphery. The MrgprB4^+^ cells responded to innocuous mechanical stimuli designed to mimic stroking and allogrooming, but not to rapid noxious mechanical stimuli. Thus, MrgprB4 likely marks a class of C fibers that are tuned to gentle mechanical stimuli first described in humans in the 1930s (Zotterman, 1939).

In contrast, the same imaging approach was employed to demonstrate that MrgprD^+^ neurons respond only to pinching but not stroking stimuli. Thus, the expression of these two Mrgpr genes nicely differentiates C fiber subtypes tuned to different mechanical stimuli. Moreover, activation of these different C fiber types likely conveys different valences (pleasurable/calming vs. noxious/alarming). Interestingly, the MgrprB4^+^ neurons could only be activated via mechanical stimulation at the periphery in an intact preparation, but not in an *ex vivo* skin-nerve preparation, whereas MrgprD^+^ cells could be activated in either type of preparation. The reason for this discrepancy is unknown, although the authors hypothesize that intact hair as well as underlying connective tissue might be important to the detection of the stroking stimulus, which is more spatially distributed, whereas the MrgprD cells respond to a much more narrowly applied stimulus (e.g., von Frey stimulation).

To understand the behavioral consequence of activating MrgprB4^+^ neurons, the authors sought to selectively excite these neurons. To achieve pharmacological control, they used the same MrgprB4-tdTomato-2A-Cre line and viral approach to selectively express DREADD, a GPCR engineered to be activated by the synthetic ligand CNO that is not naturally produced in the mice (Lee et al., 2013). The authors examined the consequences of inducing DREADD activity in either MrgprB4^+^ or MrgprD^+^ neurons in behaving mice. In a conditioned place preference (CPP) paradigm, the initial preference of mice for one chamber was reversed in favor of another that was associated with CNO (i.e., activation of the MrgprB4^+^ neurons) during conditioning. This suggested that sensation mediated by MrgprB4^+^ cells has a positive affective valence. However, since this was the only behavior test used to examine the valence question in these mice, further testing would be an informative and valuable area of investigation. Furthermore, activation of MrgprD^+^ cells in the same CPP assay promoted neither preference nor aversion. The latter result is somewhat surprising, as one might have expected that activation of these nociceptors would have a strong negative valence.

## Molecular dissection of other C fibers tuned to low threshold mechanical stimuli (C-LTMRs)

There exist other classes of C fibers that respond to low-threshold stimuli (C-LTMRs), distinct from those described above that express MrgprB4 and those that respond to high threshold stimulation and express MrgprD. Two markers have recently been described as selectively labeling unique classes of C-LTMRs in mice, the vesicular glutamate transporter type 3 (VGluT3; Seal et al., 2009) and tyrosine hydroxylase (TH; Li et al., 2011).

Glutamatergic neurons rely on transporters to load the glutamate into synaptic vesicles. Three such transporters exist in mice (VGLUT1-3) and are differentially expressed (Fremeau et al., 2004). To examine the distribution of VGLUT3, a BAC transgenic strain was created to express EGFP under the VGluT3 promoter (Seal et al., 2009). Examination of these mice revealed that, in the periphery, VGLUT3 is expressed in a subpopulation of small-diameter somatosensory neurons. VGLUT3^+^ neurons are unmyelinated, non-peptidergic neurons. These neurons project to spinal cord laminae I and II and account for roughly 10% of L4/L5 DRG and trigeminal neurons. They do not coexpress TRPV1 and only a small percentage (7%) bind IB4. VGlut3 is also found in the cochlea and regions of the CNS (Fremeau et al., 2002; Gras et al., 2002; Seal et al., 2008).

In a separate study, genetic examination of the TH^+^ sensory neuron population revealed this gene is also expressed in unmyelinated C fibers that are non-peptidergic, do not express any of the TRP channels or Mrgprs characterized to date, and do not bind IB4 (Li et al., 2011). However, there is significant overlap in expression between VGLUT3 and TH, suggesting that the expression of these two genes mark a similar population of C-LTMRs (Li et al., 2011). To visualize the axonal endings of C-LTMRs in the skin, Li et al. (2011) crossed the TH-CreER mouse line to two reporter strains. C-LTMRs exclusively innervated hairy but not glabrous skin. In addition, an individual C-LTMR axon branch was found to arborize extensively into longitudinal lanceolate endings, associating with about 18 hair follicles in the back skin of the mouse. These results correlate well with studies in humans that also found that physiologically defined C-tactiles exclusively innervate hairy skin (Olausson et al., 2002).

*Ex vivo* recordings from an intact preparation of thoracic spinal cord, DRG, nerves, and skin from the VGLUT3-EGFP mice showed that VGLUT3^+^ sensory neurons are exquisitely sensitive to mechanostimulation (i.e., 0.07 mN von Frey stimulus; Seal et al., 2009). They respond better to slowly moving stimuli than to rapidly moving ones and adapt to stationary stimuli. Finally, the authors found that these neurons respond to cooling but not heating stimuli. These physiological attributes are nearly identical to those found in TH^+^ C-LTMRs, again supporting the notion that these are largely the same class of neurons (Li et al., 2011).

Since VGLUT1 and 2 are not co-expressed with VGLUT3 in somatosensory neurons, VGLUT3-knockout neurons fail to release glutamate and excite their downstream synaptic partners. VGLUT3-knockout mice were found to be essentially normal in their sensitivity to cold, heat, formalin, and von Frey stimuli (Seal et al., 2009). However, when challenged in chronic pain paradigms (intraplantar carrageenan injection; spared nerve injury; hindpaw incision; capsaicin injection into the ankle), compared with wild-type littermates, these mice were protected from mechanical but not from temperature hypersensitivity. In addition, given their sensitivity to low threshold stimuli as demonstrated by the skin-nerve recordings, it seems likely that VGluT3^+^ sensory neurons have functions other than sensing noxious mechanical stimuli. Moreover, the widespread expression of VGluT3 throughout the CNS confounds interpretation of these behavioral experiments (Fremeau et al., 2002; Gras et al., 2002). And in contradiction with this result, the work of Lou et al. (2013) has shown that Runx1 (runt domain transcription factor 1) knockout mice, which lack proper development of VGluT3 neurons, have unimpaired acute and chronic mechanical pain. Ultimately, conditional knockout of VGLUT3 solely in sensory neurons will be required to resolve this discrepancy.

## Markers for additional classes of touch neurons

In addition to the TH^+^ C-LTMRs described above, mouse lines have also been described that selectively label Aδ-LTMRs and Aβ-RA-LTMRs (rapidly-adapting LTMRs; Li et al., 2011). The authors characterized both the peripheral and central innervation patterns of each of these LTMR subtypes. In a search for additional markers, the authors generated tyrosine receptor kinase B (TrkB-tauEGFP) knock-in mice to test the hypothesis that the Aδ-LTMRs are TrkB^+^. Interestingly, TrkB expression is especially dense in thoracic-level DRGs but very low at limb levels (whether cervical or lumbar). TrkB^+^ neurons lacked markers of mechanosensitive C fibers such as TH and IB4. Critically, these TrkB^+^ neurons exhibited all the characteristic properties of Aδ-LTMRs in an *ex vivo* skin-nerve preparation, including extreme mechanical sensitivity (i.e., von Frey threshold <0.07 mN), rapidly adapting responses to suprathreshold stimuli, intermediate conduction velocities, and sensitivity to cooling but not warming of the skin. Similar to the TH^+^ C-LTMRs, the TrkB^+^ Aδ-LTMRs form lanceolate endings associated with hair follicles of the trunk.

Li et al. (2011) identified the neuropeptide Y receptor type 2 (Npy2r-GFP) mouse line by screening through the available BAC-GFP GENSAT lines for the expected projection pattern of Aβ-LTMR neurons to laminae III through V of spinal cord dorsal horn (Gong et al., 2003). The Npy2r-GFP cells account for 6% of thoracic DRG neurons. They are heavily myelinated large-diameter cells that co-express a marker for myelinated sensory neurons (neurofilament heavy chain 200 or NF200) and lack expression of C- and Aδ-LTMRs markers TH or TrkB, respectively. They also have the electrophysiological hallmarks of the rapidly adapting subtype of Aβ-LTMRs, Aβ-RA-LTMRs, characterized by low mechanical threshold (0.07 mN), rapid conduction velocities, and rapidly adapting responses. All of the peripheral endings of Npy2r-GFP^+^ cells form longitudinal lanceolate endings.

In an informative and complex series of matings, the authors managed to cross various combinations of three lines of labeled LTMRs (TH^+^, TrkB^+^, and Npy2r^+^) thus allowing them to visualize the organization of cutaneous endings of the different neuron types relative to each other. This painstaking investigation affords us a glimpse into the complexity of the touch-sensitive detection organs innervated by LTMRs in the skin as well as a nascent appreciation of the organization of sensory neuron projections in the spinal cord.

These crosses revealed that each of the three types of hair follicles in mouse (guard, awl/auchene, and zigzag) is a neurophysiologically distinct mechanosensory organ, innervated by different combinations of mechanosensory fiber types. Zigzag hair follicles are innervated by both C- and Aδ-LTMRs; guard hairs are innervated only by Aβ-LTMRs; and awl/auchene hairs are triply innervated by all three fiber types. Furthermore, crossing the TH-reporter mice with the TrKB-reporter mice demonstrated that the lanceolate endings of C- and Aδ-LTMRs were interdigitated with one another around the same hair follicles. This comingled spatial organization correlates with their functional similarities, since both neuron types respond to very light touch (0.07 mN) as well as to rapid cooling.

When the authors examined the central projections of the different neuron classes in the spinal cord, they observed that each class projected to a distinct but overlapping termination zone in the dorsal horn. C-LTMRs terminated within lamina II; Aδ-LTMRs within lamina II and III, and Aβ-RA-LTMRs within layers III-V. Retrograde labeling of cutaneous sensory neurons revealed a columnar organization within the spinal cord, such that LTMR neurons innervating the same patch of skin project to a spatially restricted column in the spinal cord that is perpendicular to the laminae, extending from laminae II through V in concordance with the termination zone of each neuron class.

When two different labels were retrogradely injected in patches of skin in close proximity, the projections of the labeled neurons were found to be near each other in spinal cord, revealing a somatotopic organization. Thus, using genetic-labeling strategies (combined, for some experiments, with viral expression), the authors were able to derive insights into spinal cord processing of light touch in much the same manner as had been employed using TRP channels and Mrgprs for temperature, nociception, and itch. In addition, the divisions between the subclasses of LTMRs appear to be preserved in the spinal cord, with maintenance of the segregation between neuron subtypes within the central projections. It will be extremely interesting to examine these three types of LTMRs functionally and behaviorally in a paradigm similar to that published on for MrgprB4 and MrgprD (Vrontou et al., 2013).

Another group has searched for markers of specific subtypes of trigeminal ganglion touch neurons innervating the whiskers. Sakurai et al. (2013) characterized chondrolectin-PLAP mice as well as parvalbumin-Cre mice crossed to Cre-dependent reporter lines. They found that chondrolectin labels RA touch neurons innervating the mystacial pad with lanceolate endings. In contrast, parvalbumin labeled two types of touch neurons: SA (slowly adapting) neurons with Merkel endings, and a small number of RA neurons with longitudinal lanceolate endings. By crossing the two lines together, the authors found that 25% of parvalbumin^+^ cells in the TG coexpress chondrolectin, and that 22% of the chondrolectin^+^ neurons also express parvalbumin (Sakurai et al., 2013).

## Beyond cell types: using transgenic mice to study important drug targets

While the focus of this review has been on markers that narrowly define specific subpopulations, several key molecules involved in somatosensory function span multiple sensory neuronal classes. Opioids have long been known to exert analgesic effects and alleviate pain, however, it was unclear how and where this was taking place since opioid receptors are present both centrally and peripherally. Furthermore, in the periphery, mu and delta opioid receptors (MOR and DOR, respectively) had previously been thought to be co-expressed within the same C fibers, confounding the ability to parse out their relative contributions (Gomes et al., 2004).

**Figure 5:**
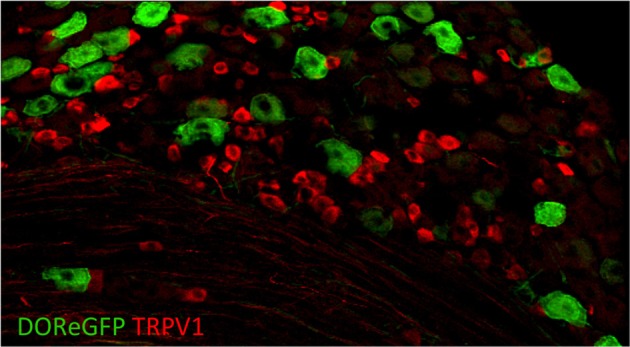
**Delta opioid receptor (DOR-eGFP mice, green) rarely overlaps with TRPV1 (red)**. Image courtesy of Grégory Scherrer, Stanford University.

To better understand the expression of opioid receptors in the peripheral nervous system, Scherrer et al. (2009) created a mouse where the DOR coding region was fused to EGFP. With these mice, the authors were able to clarify the actual distribution of these important receptors within sensory neurons. Quite surprisingly, they found that DORs are only expressed in about 17% of DRGs and that MORs and DORs are almost never co-expressed in the same neurons but rather segregate into different classes of C fibers. DOR mostly does not overlap with TRPV1 (Figure 5) but rather is found in roughly half of the myelinated fibers and about a third of the non-peptidergic C fibers. Such a distribution contrasts with that of MOR, which is expressed mainly in TRPV1^+^ neurons. More recently, Bardoni et al. (2014) have confirmed the expression pattern observed in the DOR-EGFP mouse, using an ultrasensitive *in situ* hybridization method as well as radioligand binding studies. They also further investigated MOR and DOR coexpression and found that fewer than 5% of DRG neurons coexpress both opioid receptors, with most of these cells being NF200^+^ CGRP^+^ nociceptors (~88%), and the rest being NF200^−^ IB4-binding MrgprD^+^ mechanoreceptors or peptidergic TRPV1^+^ thermonociceptors.

Based on this expression pattern, one might thus expect a role for DORs in mechanical sensitivity vs. one for MORs in heat sensitivity. Using pharmacological inhibition of each type of opioid receptor in combination with ablation of TRPV1^+^ neurons in models of inflammatory and neuropathic pain, Scherrer et al. (2009) indeed found that MORs selectively contribute to heat pain while DORs contribute to mechanical pain. In this way, the authors were able to show that the two receptors selectively regulate mechanical vs. heat hypersensitivity. This mouse thus provides insight into the potential for modality-specific opioid drug targets, and the development of novel pain therapeutics. Beyond the periphery, the central mechanisms of opioid function remains an area that still requires further elucidation.

It should be mentioned that the expression pattern of DOR in the DRGs is a matter of some controversy, since another group has obtained quite different results using double *in situ* hybridization, immunohistochemistry with multiple antibodies, single-cell PCR, and electrophysiology (Wang et al., 2010; He et al., 2011). This group found that a much larger population of sensory neurons expresses DORs (~70–80%), and that these comprise both large and small diameter peptidergic neurons. Furthermore, they observed that DORs and MORs are coexpressed in small peptidergic neurons (73% of MOR^+^ neurons are also DOR^+^). Reconciling these findings will perhaps require the generation of another labeled mouse line, to test whether the findings in the DOR-EGFP line can be reproduced by another group. Overall, one would have expected the genetic method to be more reliable than other expression analyses.

## Beyond the periphery: revealing central mechanisms of integration of somatosensory information with transgenic mice

Although the references cited throughout our review initially focused on defining markers of various sensory neuron types, these studies also derived new insights into the central integration of somatosensory information, sometimes based on the anatomy of spinal cord projections alone. These studies have set the stage for a deeper appreciation of the neural circuits that encode information from the periphery.

For example, a major finding of the work of Li et al. (2011) on C-LTMRs is that touch neurons of different types that innervate the same small patch of hairy skin all project to a spatially circumscribed column in the spinal cord, with different LTMR types projecting to distinct but overlapping bands of the column. An additional finding from this study is the existence of a somatotopic map in the spinal cord, such that neurons of the same type innervating neighboring patches of skin project to neighboring columns in the spinal cord. Thus, despite comingling of cell bodies belonging to different LTMR neuron classes in the DRG, the columnar and somatotopic organization of their central projections suggest the spinal cord is a likely integration site for mechanosensory information. Another region that would be interesting to examine in these mice in future studies would be the organization of projections to the dorsal column nuclei, a region of the brainstem known to be important for the sensation of touch.

Similarly, the CGRP^+^ neuron ablation study revealed aspects of central processing, some of which are still not fully understood (McCoy et al., 2013). First, the phenotype of mice lacking CGRPα^+^ neurons was greater than anticipated, showing that CGRPα^+^ neurons likely contribute not only to heat sensitivity, but also indirectly to the perception of cold, by providing tonic inhibition to TRPM8 inputs. Thus, in addition to providing valuable information on the modalities detected by peptidergic C fiber afferents, this mouse line also points to crosstalk between modalities in the spinal cord, as somatosensory information from one population of neurons (e.g., TRPM8^+^) appears to be modulated by another (CGRP^+^). How widespread this phenomenon is remains to be addressed. Second, and likely relating to more central mechanisms, the authors also reported further intriguing phenotypes of these mice, possibly related to the cold hypersensitivity phenotype. The coats of DTX-treated mice were piloerect and their core temperature dropped lower than wild type mice in response to immersion in warm water. The DTX-treated mice also weighed less than saline-treated counterparts, suggesting a role for CGRPα^+^ neurons in metabolism. Indeed, perturbation of core temperature regulation has also been demonstrated following pharmacological activation and inhibition of TRPV1 and TRPM8 (Patapoutian et al., 2009). How thermosensitive sensory neurons modulate metabolism remains a central mystery yet to be elucidated.

## Summary and future directions

The somatosensation field has benefitted immensely from the identification of a number of key genes required for the signaling of distinct sensory modalities (for example heat, cooling, pain, and itch). These discoveries have led to remarkable mechanistic insights into how sensory stimuli are detected, and have also provided neuroanatomists and geneticists with key molecules and loci that can be used to identify and probe the function of cohorts of specialized sensory neurons (Figure 4). Indeed, the discovery of a panel of ion channels and somatosensory receptor genes has presented researchers with genetic loci with which to selectively investigate and manipulate somatosensory neurons (Table 2). Gene-targeting strategies have been used to introduce histological markers (such as GFP, LacZ, and/or PLAP) that have allowed for the tracking of neuronal projections with single process resolution (i.e., Figure 3). Beyond anatomy, these loci have been used to express toxin receptors for cell ablation or Cre recombinases that enable ectopic expression of gene silencers/activators with a high degree of cell-type specificity. These studies have allowed researchers to probe the function of specific subtypes of somatosensory neurons with unprecedented resolution.

### How many subtypes exist?

While great progress has been made, we still do not know how many distinct subtypes exist. Indeed, further molecular dissection of the sensory neurons classes seems likely to continue to advance our understanding. In the case of thermosensation, we are still ignorant of the processes by which attraction to warm stimuli vs. aversion to noxious thermal stimuli are encoded. The TRPV1 population seems to have a particularly diverse set of functions that remain to be parsed out. TRPV1^+^ neurons are required for the detection of heat, irritants, and pruritogens, but exactly how these distinct sensations arise remains unknown. One population that clearly would benefit from genetic labeling is the group of TRPA1-expressing neurons. TRPA1 is expressed in a subset of TRPV1^+^ neurons (Jordt et al., 2004). Activation of TRPA1 is required for the response to environmental irritants such as mustard oil (Bautista et al., 2006). However, TRPA1 is also required for the detection of classes of pruritogens such as chloroquine (Wilson et al., 2011). These two chemicals clearly elicit unique sensations (stinging vs. itch) and thus hint at further, yet to be discovered, neuronal specializations. Unfortunately, no TRPA1 reporter mice or Cre strains are available as of yet.

Another unique class of TRPV1^+^ neurons seems to be those that co-express CGRP. The profound thermal deficits in the CGRP^+^ neuron ablation studies are quite surprising, given that half of the TRPV1^+^ neurons are preserved (McCoy et al., 2013). What is the function of the remaining TRPV1^+^ neurons? Along similar lines, what is the role of the TRPV1^+^ neurons that also express TRPM8 (Takashima et al., 2007; Dhaka et al., 2008)? There would seem to be little logic for co-expression if the goal was temperature discrimination. Perhaps the TRPV1/TRPM8 neurons are general noxious temperature sensors?

Beyond thermosensation, our understanding of touch transduction is still in its infancy. As the molecules involved in mechanotransduction are discovered, they will surely offer exciting prospects for genetic manipulation of these classes of neurons. For example, the identification of the putative mechanosensitivity channel-like protein Piezo 1 and 2 offers yet another ripe genetic target to study touch neurons (Coste et al., 2010). More broadly, advances in molecular profiling of both RNA and protein make it safe to predict new markers are on the horizon.

### What is happening downstream of the sensory neurons?

As exciting as they are, the studies described above are in some ways just the preamble to the much larger task of understanding how the brain encodes somatosensory information. Knowing there exist classes of neurons that specifically encode cold, heat, and gentle touch presents a groundbreaking opportunity to begin unraveling the downstream pathways. The manipulation of sensory neuron populations has already begun to yield insights into the potential organization of neural circuits that receive sensory neuron input. For example, the ablation of CGRP^+^ neurons showed that they normally provide tonic inhibition to TRPM8^+^ neurons, revealing crosstalk between different modalities (McCoy et al., 2013). Similarly, the investigation of Bhlhb5-knockout mice revealed that this transcription factor is required for the survival of a population of spinal interneurons that regulate itch via inhibition (Ross et al., 2010). Furthermore, deletion of the testicular orphan nuclear receptor 4 (TR4) leads to the loss of a population of excitatory interneurons and a dramatic insensitivity to itch and pain (Wang et al., 2013). Finally, another transcription factor knockout study (Tlx3) finds that lack of excitatory interneurons in the dorsal spinal cord dramatically alters nocifensive behaviors to a wide variety of stimuli (Xu et al., 2013).

The transgenic strains coupled with those developed for studying the CNS will allow further access to technologies for circuit mapping. Optogenetic techniques have been catalyzing advances throughout neuroscience, and there is no reason to believe the same will not hold true for somatosensory research (Madisen et al., 2012). For example, targeting channelrhodopsin (ChR) to specific sensory neuron classes will allow selective activation of neurons that currently lack defined pharmacology. Indeed, ChR2 has already been targeted to nociceptors where it was found to be sufficient to drive nocifensive behaviors (Daou et al., 2013). Similarly, the use of chemical genetics, such as DREADDS, have also proved valuable in dissecting the behavioral contributions of defined sensory neuron populations (Lee et al., 2013; Vrontou et al., 2013). Finally, such reagents will hopefully result in a better understanding of the neural circuits that receive input from sensory neurons. For example, by expressing ChR2 in MrgprD^+^ neurons, Wang and Zylka (2009) have characterized the synaptic partners of this class of sensory neurons in lamina II, and detected distinct functional modules in the spinal cord. The stage is now set for subsequent studies to capitalize on the current knowledge of the different classes of somatosensory neurons coupled with opto- and chemical genetic techniques. The circuits and functions of the somatosensory system have thus become a ripe area for future investigation.

### Conflict of interest statement

The authors declare that the research was conducted in the absence of any commercial or financial relationships that could be construed as a potential conflict of interest.
